# Tau phosphorylation affects its axonal transport and degradation

**DOI:** 10.1016/j.neurobiolaging.2013.03.015

**Published:** 2013-09

**Authors:** Teresa Rodríguez-Martín, Inmaculada Cuchillo-Ibáñez, Wendy Noble, Fanon Nyenya, Brian H. Anderton, Diane P. Hanger

**Affiliations:** Department of Neuroscience, King's College London, Institute of Psychiatry, London, UK

**Keywords:** Tau phosphorylation, Axonal transport, Degradation

## Abstract

Phosphorylated forms of microtubule-associated protein tau accumulate in neurofibrillary tangles in Alzheimer's disease. To investigate the effects of specific phosphorylated tau residues on its function, wild type or phosphomutant tau was expressed in cells. Elevated tau phosphorylation decreased its microtubule binding and bundling, and increased the number of motile tau particles, without affecting axonal transport kinetics. In contrast, reducing tau phosphorylation enhanced the amount of tau bound to microtubules and inhibited axonal transport of tau. To determine whether differential tau clearance is responsible for the increase in phosphomimic tau, we inhibited autophagy in neurons which resulted in a 3-fold accumulation of phosphomimic tau compared with wild type tau, and endogenous tau was unaffected. In autophagy-deficient mouse embryonic fibroblasts, but not in neurons, proteasomal degradation of phosphomutant tau was also reduced compared with wild type tau. Therefore, autophagic and proteasomal pathways are involved in tau degradation, with autophagy appearing to be the primary route for clearing phosphorylated tau in neurons. Defective autophagy might contribute to the accumulaton of tau in neurodegenerative diseases.

## 1 Introduction

The microtubule-associated protein tau is a cytoskeletal protein expressed primarily in the central nervous system where it stabilizes microtubules and regulates neurite outgrowth. Several neurodegenerative diseases, collectively termed tauopathies, are characterized by intraneuronal inclusions of straight or paired helical filaments of insoluble, highly phosphorylated tau (Ballatore et al., 2007; Gallo et al., 2007). Interaction of tau with microtubules occurs primarily through the repeated microtubule-binding domains located in the C-terminal half of tau. The inclusion of either 3 or 4 binding repeats is regulated by alternative splicing of exon 10, which gives rise to either 3R or 4R tau isoforms (Goedert et al., 1989). Tau also interacts with components of the plasma membrane through its amino terminal projection domain (Brandt et al., 1995; Gauthier-Kemper et al., 2011; Pooler et al., 2012). Phosphorylation of tau decreases its capacity to bind to and stabilize microtubules (Bramblett et al., 1993; Busciglio et al., 1995), and recently we have found that association of tau with the plasma membrane is also regulated by phosphorylation (Pooler et al., 2012).

In Alzheimer's disease, tau is highly phosphorylated, and many of the tau phosphorylation sites targeted by glycogen synthase kinase-3 (GSK-3), the primary candidate kinase for tau phosphorylation, are also phosphorylated in insoluble tau isolated from Alzheimer brain (Avila et al., 2012; Hanger et al., 2007, 2009). Changes in protein phosphorylation have been reported to affect axonal transport in models of neurodegenerative diseases. For example, increased phosphorylation of neurofilaments and the microtubule-binding protein, MAP1B, reduces their respective rates of axonal transport (Ackerley et al., 2003; Ma et al., 2000). In contrast, elevated phosphorylation of tau results in an increased overall slow rate of tau transport in neurons, and inhibiting GSK-3 phosphorylation of tau reduces its motility (Cuchillo-Ibanez et al., 2008). Defective axonal transport has been suggested to underlie some forms of neurodegenerative disease (De Vos et al., 2008; Falzone et al., 2010), although the mechanisms responsible are not established.

Because, in addition to affecting its transport, tau phosphorylation affects its ability to be degraded (Ding et al., 2006; Johnson, 2006), we examined the contributions of the ubiquitin-proteasome system (UPS) and of macroautophagy (autophagy) to tau degradation in the context of tau transport. Though the UPS eliminates short-lived proteins that are targeted for degradation by the addition of ubiquitin, the autophagic system removes long-lived, structural proteins and/or damaged or misfolded proteins (Martinez-Vicente and Cuervo, 2007; Shacka et al., 2008). Furthermore, a particular form of autophagy, termed aggrephagy, eliminates accumulated and aggregated ubiquitinated proteins, such as those deposited in the brain in the tauopathies and therefore, this process is gaining particular interest in neurodegenerative disease (Yamamoto and Simonsen, 2011). Dysfunction of autophagy and the UPS in neurons has been linked to a number of neurodegenerative disorders characterized by accumulation of misfolded protein aggregates (Bauereis et al., 2011; de Vrij et al., 2004; Li et al., 2010; Martinez-Vicente and Cuervo, 2007; Riederer et al., 2011; Tai and Schuman, 2008). Tau degradation by each of these processes has been the subject of extensive research. However, though some studies suggest that tau is not a substrate of the proteasome (Brown et al., 2005; Delobel et al., 2005; Feuillette et al., 2005), others have reported that inhibition of the UPS results in tau accumulation in cells (David et al., 2002; Liu et al., 2009; Zhang et al., 2005). Autophagy has also been shown to degrade wild type and modified forms of tau, including caspase-cleaved and C-terminally truncated species (Dolan and Johnson, 2010; Wang et al., 2010). However, induction of autophagy with rapamycin in adult rat brain does not result in proteolysis of endogenous tau, suggesting that this lysosomal system might not participate in physiological tau degradation (Zhang et al., 2009).

In this study, we have used mutant forms of tau, which mimic permanent states of phosphorylation and dephosphorylation, to investigate tau function. In particular, we have examined the effect of tau phosphorylation on its rate of axonal transport and its degradation by the UPS and autophagy. We show here that phosphomimic tau binds less to microtubules and exhibits an increased number of motile tau particles in axons compared with wild type tau. We also show that, in primary cortical neurons, exogenously expressed phosphomimic and wild type tau are targets for autophagy, and endogenous tau is not. In the absence of autophagy, degradation of tau by the UPS in neurons is minimal.

## 2 Methods

### 2.1 Plasmids

Plasmids expressing the longest human central nervous system tau isoform (2N4R; WTtau) and 2 tau phosphomimic mutants, E18tau and E27tau, with 18 or 27 serine/threonine residues mutated to glutamate, respectively, were gifts from M. Goedert (MRC Laboratory of Molecular Biology, Cambridge, UK). The tau sequences were cloned into the enhanced green fluorescent protein (EGFP)-C1 vector (BD Biosciences, Clontech) to generate a construct in which EGFP was fused to the amino terminus of tau. Site-directed mutagenesis (Quikchange kit, Stratagene) was used to create an additional EGFP-tagged tau mutant construct encoding A18tau in which the same 18 serine/threonine residues that were mutated in E18tau were substituted by alanine (Cuchillo-Ibanez et al., 2008). Thus, A18tau represented a mimic of permanent dephosphorylation at these sites in tau because it cannot be phosphorylated at these 18 residues. The E27tau sequence was also cloned into the DsRed fluorescent protein vector (BD Biosciences, Clontech). The plasmid expressing microtubule-associated protein 1 light-chain 3 (LC3), fused to EGFP, was a gift from A.M. Tolkovsky (University of Cambridge, UK).

### 2.2 Cell culture and transfection

Embryonic day 18 (E18) rat cortical neurons were cultured in Neurobasal medium (Life Technologies, Inc), supplemented with B27 (Invitrogen), as described previously (Cuchillo-Ibanez et al., 2008). Chinese hamster ovary (CHO) cells were cultured in Ham's F12 and mouse embryonic fibroblasts (MEFs) in Dulbecco's Modified Eagle's Medium, each containing 10% (vol/vol) fetal calf serum. All culture media were supplemented with 100 U/mL penicillin, 100 μg/mL streptomycin, and 2 mM L-glutamine. Cortical neurons were transfected using either calcium phosphate (Promega) for live microscopy or Lipofectamine 2000 (Invitrogen) for analysis of cell lysates. CHO cells and MEFs were transfected using Lipofectamine (Invitrogen), following the manufacturers' instructions.

### 2.3 Time-lapse imaging of transfected neurons

E18 rat cortical neurons (5 days in vitro [DIV]) were transfected with plasmids expressing WTtau, E18tau, E27tau or A18tau, and time-lapse images were recorded after 48 hours. Coverslips were placed in a sealed chamber with Neurobasal medium containing B27 and maintained at 37 °C with 5% CO_2_ perfusion on the stage of an Axiovert 200M Zeiss microscope. Epifluorescence illumination was attenuated to 8% using neutral density filters. Images were collected at 1–2-second intervals for 2–4 minutes using a Plan-ApoChromat 100× 1.4 NA oil immersion objective (1 pixel = 0.063 μm), a green fluorescent protein filter set and an AxioCam MRm camera. Movement of fluorescent particles was analyzed using AxioVision software 4.6.3 (tracking module). Fluorescent particles that moved <0.3 μm between consecutive frames were considered to be pausing (typically corresponding to a rate of <0.2 μm/s). Statistical analyses were performed using a 2-tailed Student *t* test.

### 2.4 Immunocytochemistry and fluorescence microscopy

Transfected CHO and MEF cells were fixed in 4% (wt/vol) paraformaldehyde 24 hours after transfection. All incubations were carried out at room temperature. Cells were blocked using 1% (wt/vol) Triton X-100, 10% (vt/vol) fetal calf serum in phosphate buffered saline (PBS) for 20 minutes, and then stained for α-tubulin (mouse monoclonal, DM1A, Invitrogen) for 1 hour. The anti-mouse Texas Red-coupled secondary antibody was added for 1 hour in the dark. Nuclear staining was performed using Hoescht 33342 (5 μg/mL bisbenzimide in PBS). Fluorescence microscopy was performed using an Axioskop microscope (Zeiss), equipped with a camera (CoolSnap HQ, Photometrics) and with Plan-NeoFluor 20× 0.50 NA, 40× 0.75 NA, and 100× 1.30 NA objectives.

### 2.5 Gel electrophoresis and Western blot analyses

For protein analysis, 6-well plates containing 1 × 10^6^ neurons per well, were rinsed with PBS at 4 °C and cells were scraped into hot (2×) Laemmli sample buffer. Proteins were separated on 10% (wt/vol) sodium dodecyl sulfate (SDS)-polyacrylamide gels and transferred to nitrocellulose membranes. Membranes were probed with antibodies to tau (rabbit polyclonal, DAKO or TP70), β-actin (mouse monoclonal, clone AC15, Sigma), S6 (mouse monoclonal, clone 54D2, Cell Signaling Technology) or P-S6 (mouse monoclonal, clone 2F9, Cell Signaling Technology). For LC3 analysis, proteins were separated on 15% (wt/vol) gels and blots were probed with LC3 antibody (rabbit polyclonal, Sigma). Antigens were visualized using secondary antibodies coupled to infra-red dyes and an Odyssey scanner (Li-Cor Biosciences).

### 2.6 Microtubule binding assay

Assays for microtubule binding of tau were performed as described previously (Ding et al., 2006), with some modifications. Briefly, CHO cells were transfected for 24 hours with plasmids expressing EGFP-tagged WTtau, E18tau, E27tau, or A18tau. Cells were rinsed with warm PBS and suspended in warm PIPES buffer (80 mM piperazine-N,N′bis-2-ethanesulfonic acid, pH 6.8, 1 mM guanosine-5′-triphosphate, 1 mM MgCl_2_, 1 mM ethylene glycol-bis(2-aminoethyl)-N,N,N′,N′-tetraacetic acid, 0.5% (wt/vol) Triton X-100 and 30% (vol/vol) glycerol), containing 1 mM phenylmethylsulfonylfluoride, Complete protease inhibitor (Roche), 0.5 μM okadaic acid (Calbiochem), and 10 μM taxol (Sigma). Cell suspensions were centrifuged at 5000*g* for 10 minutes at room temperature and an aliquot of the supernatant was retained as the postnuclear lysate (input). The remaining postnuclear lysate was centrifuged at 100,000*g* for 1 hour at room temperature. The supernatant (unbound) was retained and the pellet (microtubule-bound) was rinsed twice and resuspended in PIPES buffer. The proteins in each fraction were separated on 10% (wt/vol) SDS-polyacrylamide gels and blots were probed with the tau polyclonal antibody (DAKO).

### 2.7 Immunoprecipitation

Rat cortical neurons (5 DIV) were transfected with constructs expressing EGFP-tagged WTtau or phosphomutant tau. After 48 hours, neurons were lysed and EGFP-tau was immunoprecipitated using an agarose-conjugated polyclonal antibody to GFP (Santa Cruz Biotechnology, Inc), as described previously (Cuchillo-Ibanez et al., 2008). Immunoprecipitated proteins were probed on Western blots, using antibodies recognizing kinesin heavy chain (MAB1614, Chemicon) and tau (DAKO).

### 2.8 Glutathione-S-transferase binding assay

Purified recombinant WTtau and E27tau proteins were prepared as described (Utton et al., 1997). Glutathione-S-transferase (GST) fusion proteins were prepared and GST binding was assayed as described previously (Usardi et al., 2011). An equimolar amount of purified GST-kinesin light chain 1 or 2, bound to glutathione Sepharose 4B beads, was incubated with purified recombinant human tau in modified RIPA buffer (20 mM Tris-HCl, pH 7.4, containing 150 mM sodium chloride, 10 mM sodium fluoride, 1 mM sodium orthovanadate, 1 mM ethylenediaminetetraacetic acid, 1 mM ethylene glycol-bis(2-aminoethyl)-N,N,N′,N′-tetraacetic acid, 0.2 mM phenylmethylsulfonyl fluoride, 1% [vol/vol] Nonidet 40, Complete protease inhibitor [Roche]) for 1 hour at 4 °C with rotation. The GST-Sepharose beads were pelleted at 500*g* for 1 minute at 4 °C, washed 3 times with modified RIPA buffer, and resuspended in Laemmli sample buffer containing 40 mM dithiothreitol. GST-bound proteins were analyzed on Western blots probed with an antibody to total tau (TP70) (Davis et al., 1995).

## 3 Results

### 3.1 Tau phosphomutants exhibit differential microtubule binding in cells

**Fig. 1:**
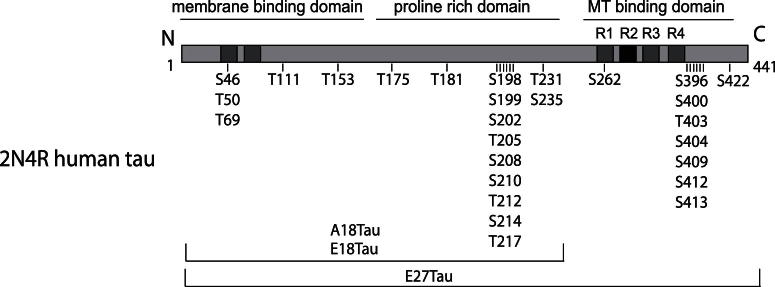
Tau phosphomutants. The diagram shows the positions in the longest human tau isoform (2N4R, 441 residues) of the serine (S) or threonine (T) residues mutated to glutamate (to mimic phosphorylation) in E18tau and E27tau, or to alanine (to preclude phosphorylation) in A18tau. R1, R2, R3, and R4 are the repeat regions of the microtubule (MT)-binding domain.

We first analyzed the interaction of wild type and phosphomutant tau with microtubules. Tau constructs included: (1) the longest human central nervous system tau isoform (WTtau, 441 residues); (2) mutant E18tau, in which serine or threonine residues are mutated to glutamate at 18 sites to mimic a permanent state of phosphorylation; (3) mutant E27tau, with 27 serine and threonine sites mutated to glutamate; or (4) A18tau, in which alanine replaces serine or threonine to preclude phosphorylation at these sites (Cuchillo-Ibanez et al., 2008) (Fig. 1).

**Fig. 2:**
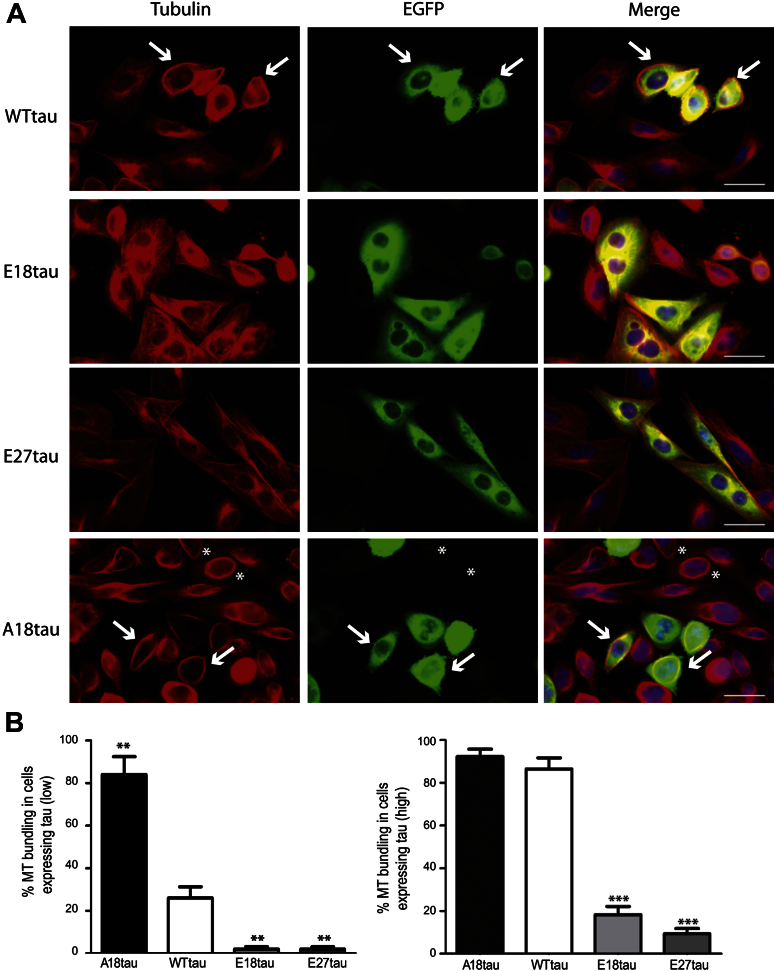
Microtubule bundling of tau constructs. (A) CHO cells were transfected with WTtau, E18tau, E27tau, or A18tau and tau expression was visualized by EGFP fluorescence. Microtubules were labeled using the monoclonal α-tubulin antibody, DM1A. WTtau and A18tau exhibited a greater ability to bundle microtubules (arrows). Asterisks indicate cells with microtubule bundles in the presence of low amounts of A18tau. Scale bar, 50μm. (B) Quantification of microtubule-bundling in cells expressing high or low amounts of tau. Values shown are mean ± standard error of the mean. ** *p* < 0.01; *** *p* < 0.001, Student *t* test, relative to WTtau. Abbreviations: CHO, Chinese hamster ovary; EGFP, enhanced green fluorescent protein; MT, microtubule.

To assess the microtubule bundling properties of each of the tau constructs used, CHO cells were transiently transfected with plasmids expressing each tau species fused to the C-terminus of EGFP (Fig. 2A). Microtubules in tau-transfected CHO cells were visualized using an antibody to α-tubulin. Twenty-four hours after transfection, cells expressing WTtau displayed widespread microtubule bundling (Fig. 2A, WTtau, arrows) with rings of tubulin apparent at the cell periphery. Cells expressing WTtau were characteristically smaller and more rounded than untransfected CHO cells. Expression of either E18tau or E27tau, the mimics of permanent phosphorylation, resulted in a dramatic reduction in microtubule bundling compared with WTtau expression in CHO cells (Fig. 2A, E18tau and E27tau). Even in highly expressing cells, microtubule bundling was rarely observed with the tau phosphomimics, and the cellular morphology was similar to that of untransfected CHO cells. In contrast, expression of the nonphosphorylatable tau mutant, A18tau, resulted in a marked increase in microtubule bundling in CHO cells, compared with WTtau (Fig. 2A, A18tau, arrows). Cells exhibiting very low amounts of A18tau also contained abundant microtubule bundles (Fig. 2A, asterisks). Similar to cells expressing WTtau, A18tau expression also resulted in cells with a smaller and more rounded morphology than their untransfected counterparts. The microtubule bundling abilities of the tau phosphomutants were quantified and expressed as the percentage of tau-expressing cells containing microtubule bundles (Fig. 2B). In cells expressing low amounts of tau, we found significant differences in the behaviors of the different phospho-tau mutants compared with WTtau (Fig. 2B; *t* test, *p* < 0.01). Low expression of A18tau resulted in bundled microtubules in 84% of the cells, compared with only 26% of the cells containing bundles with WTtau. Low expression of either of the 2 phosphomimic tau mutants resulted in negligible microtubule bundling (Fig. 2B, left). In cells expressing high amounts of tau, A18tau bundled microtubules in 92% of the cells, which was similar to WTtau (87%), and E18tau and E27tau induced bundling in only 18% and 9% of the cells, respectively. These results agree with previous findings that overexpression of wild type tau increases its binding to microtubules and induces microtubule bundling, and that these effects are reversed by increased phosphorylation (Kanai et al., 1989; Wagner et al., 1996).

**Fig. 3:**
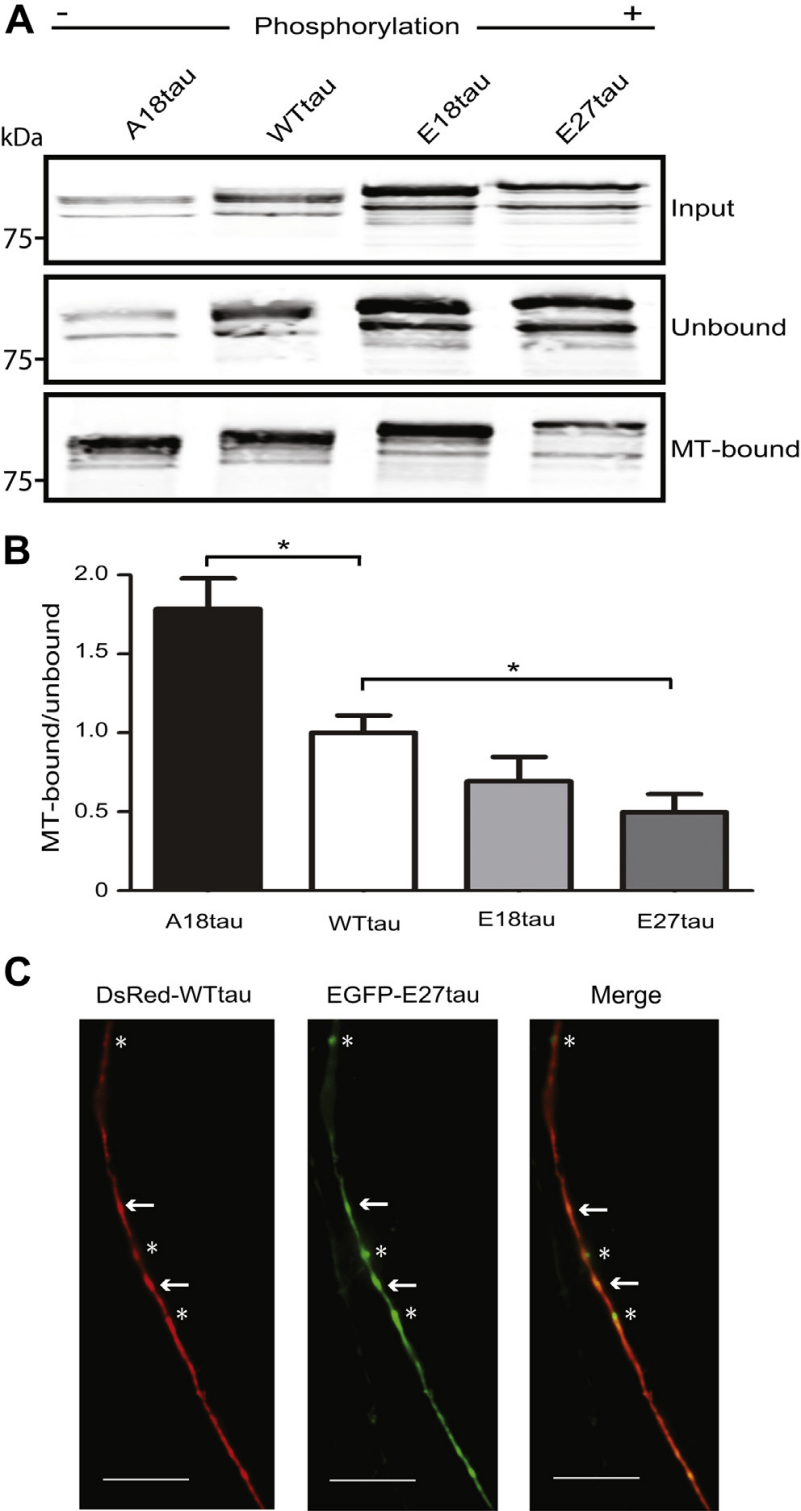
Microtubule binding of wild type and phosphomutant tau. (A) CHO cells transfected with A18tau, WTtau, E18tau, and E27tau were separated into microtubule-bound and unbound fractions and analyzed using Western blot using an antibody to tau (DAKO). (B) Quantification of microtubule-bound/unbound expressed relative to WTtau (mean ± standard error of the mean; *n* = 3); * *p* < 0.05, Student *t* test, relative to WTtau. (C) Coexpression of DsRed-tagged WTtau and EGFP-tagged E27tau in rat cortical neurons. Arrows indicate colocalization of WTtau and E27tau and asterisks indicate individual E27tau particles. Scale bar, 10 μm. Abbreviations: CHO, Chinese hamster ovary; EGFP, enhanced green fluorescent protein; MT, microtubule.

The most likely explanation for the changes in microtubule bundling that we observed is differential tubulin binding of the phosphomutant tau species. Therefore, to quantify the microtubule binding properties of different tau forms, CHO cells were transfected with constructs expressing WTtau, E18tau, E27tau, or A18tau. Forty-eight hours after transfection cells were lysed and fractionated into microtubule-bound and unbound components, based on their differential solubility (Ding et al., 2006). Comparative analysis of the cell lysates on Western blots shows that each of the tau mutants exhibits a distinct migration pattern, with E18tau and E27tau exhibiting the slowest, and A18tau the fastest, mobility on SDS-polyacrylamide gel electrophoresis (Fig. 3A). The amount of tau bound to microtubules, as a proportion of unbound tau, was expressed relative to WTtau in the transfected CHO cells (Fig. 3B). The results show that E27tau has a significantly impaired ability to bind to microtubules compared with WTtau (approximately 50%; *p* < 0.05), in agreement with earlier in vitro findings (Smith et al., 2000). E18tau also showed a trend for reduced microtubule binding of approximately 30%, but this difference was not statistically significant. In contrast, A18tau exhibited an increase of approximately 70% in its ability to bind microtubules, compared with WTtau (Student *t* test, *p* < 0.05). The results support previous findings that binding of tau to microtubules is dependent on the phosphorylation state of tau (Bramblett et al., 1993; Busciglio et al., 1995). Thus E18tau and E27tau both appear to act as functional mimics of phosphorylated tau, and A18tau as a mimic of dephosphorylated tau, with respect to binding to and bundling of microtubules (Eidenmuller et al., 2001; Leger et al., 1997; Smith et al., 2000).

Because altered microtubule binding of tau could result in its differential localization, we cotransfected rat primary cortical neurons with plasmids encoding WTtau fused to DsRed, together with EGFP-tagged E27tau. In axons of cotransfected neurons, WTtau and E27tau were widely distributed as diffuse segments and as bright puncta (Fig. 3C). Interestingly, although many WTtau puncta colocalized with E27tau (Fig. 3C, arrows), several E27tau puncta appeared to be WTtau-negative (Fig. 3C, asterisks). The reason for the apparent lack of colocalization of E27tau with WTtau throughout the axon is not clear, although it is possible that this could be because of differential microtubule binding of the 2 tau species.

### 3.2 Permanent phosphorylation does not affect the kinetic parameters of tau fast axonal transport

**Fig. 4:**
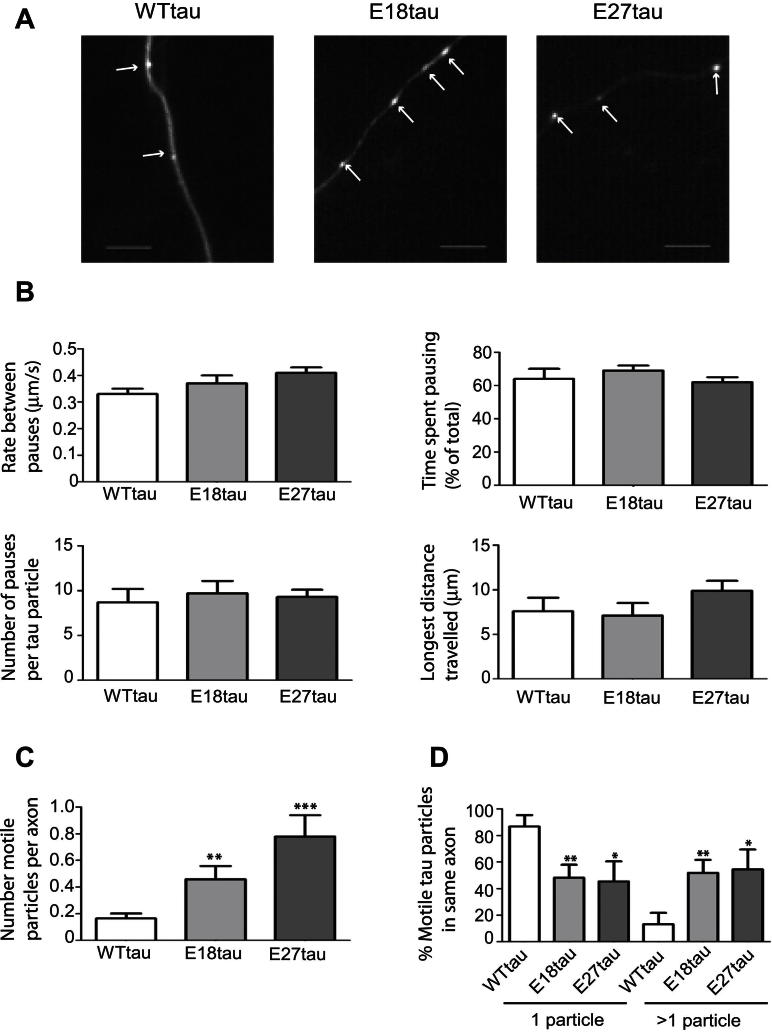
Fast axonal transport of tau. (A) WTtau, E18tau, and E27tau exhibit a punctate morphology in axons in transfected neurons. Scale bar, 5 μm. (B) Kinetic parameters of motile tau particles. Rate between pauses, time spent pausing, number of pauses, and longest distance traveled between pauses were calculated for each tau construct (WTtau, *n* = 154; E18tau, *n* = 89; E27tau, *n* = 81). (C) The number of motile particles per axon is shown for each tau construct. (D) The probability of finding 1 or more than 1 motile tau particle moving in an individual axon was calculated from a randomized selection of movies (WTtau, *n* =12; E18tau, *n* = 8; E27tau, *n* = 7). Values shown are mean ± standard error of the mean. * *p* < 0.05, ** *p* <0.01, *** *p* < 0.001, Student *t* test, relative to WTtau.

In previous studies of tau transport in neurons, we reported that axonal motility of tau is a microtubule-dependent process (Utton et al., 2005) and that E18tau and E27tau exhibited significantly increased overall rates of slow axonal transport, whereas A18tau moved more slowly than WTtau (Cuchillo-Ibanez et al., 2008). To investigate the mechanism underlying the altered rates of transport exhibited by the phosphotau mutants, we acquired time-lapse movies of individual wild type and mutant tau particles in rat cortical neurons (Fig. 4A and Supplementary Videos 1–3). The rates of fast axonal transport of each form of tau were calculated, excluding periods during which the tau particles were paused. Interestingly, no motile particles of A18tau were observed during any of the recordings made after expression in neurons and, hence, a kinetic analysis of the axonal transport of A18tau was not possible. This lack of motility might be because of the increased microtubule binding of A18tau (Fig. 3B) and hence this is a likely contributing factor to the reduced rate of overall slow transport of A18tau that we reported previously (Cuchillo-Ibanez et al., 2008).

Kinetic analysis of all moving tau particles was used to determine their rates of transport between pauses, time spent pausing, number of pauses, and longest distance traveled by each particle during the recording period (Fig. 4B). The rates of fast axonal transport of WTtau, E18tau, and E27tau were determined to be 0.33–0.41 μm/s, in line with our previous report of 0.36 μm/s for kinesin-driven transport of the human 0N4R tau isoform (Utton et al., 2005). Similar rates have been obtained for moving particles of recombinant kinesin (0.37 μm/s) (Thorn et al., 2000) and neurofilaments (0.40 μm/s) (Wang et al., 2000), consistent with kinesin-driven fast axonal transport. We could not detect any statistically significant difference between the rate of transport of WTtau and either of the 2 phosphomimic tau species. Comparison of the time spent pausing, the number of pauses, and the longest distance traveled by WTtau and phosphomimic tau also revealed no differences in these parameters. Our results indicate that alterations in tau that mimic a permanent state of phosphorylation do not affect the kinetic parameters of fast axonal transport, based on a study of individual tau particles. However, the complete lack of mobility of particles containing A18tau, suggests that lack of phosphorylation hinders fast axonal transport of tau, possibly because of stabilization of microtubules.

### 3.3 Increased motile particles of phosphomimic tau

We next examined the number of moving particles of exogenously expressed tau in neurons (Fig. 4C). A minority of axons expressing WTtau contained motile tau particles, resulting in the detection of an average of 0.16 ± 0.04 (mean ± standard error of the mean; *n* = 164) WTtau particles per axon (Fig. 4C). In contrast, increased numbers of moving particles of E18tau (0.46 ± 0.10 per axon; *n* = 106) and E27tau (0.78 ± 0.16 per axon; *n* = 100) were detected, both of which were statistically significantly different from WTtau (Student *t* test, *p* < 0.05). These results demonstrate that the frequency of motile particles of phosphomimic tau was approximately 3–5-fold greater than that of WTtau (Fig. 4C).

Examination of individual axons harboring motile tau particles showed that fewer motile particles of WTtau were present in the same axon, compared with each of the tau phosphomimics (Supplementary Videos 1–3). Thus, the probability of detecting 1 motile WTtau particle in an axon was 87%, and only 13% of axons harbored more than 1 motile particle (Fig. 4D). In contrast, approximately 50% of axons contained 1 moving particle of phosphomimic tau (Fig. 4D; Student *t* test, *p* < 0.05).

These results indicate that, based on the increased number of motile tau puncta, phosphorylation of tau significantly increases its probability of being transported in neurons and that tau axonal transport is hindered by dephosphorylation. However, the likelihood of movement of phosphomimic tau puncta did not appear to influence their kinetic parameters. Furthermore, this mechanism could help to explain in part the reduced overall rate of slow axonal transport of tau observed after tau dephosphorylation in the presence of lithium, that we observed previously with these phosphomutant forms of tau (Cuchillo-Ibanez et al., 2008).

### 3.4 Pseudophosphorylation does not affect the ability of tau to bind kinesin in neurons

**Fig. 5:**
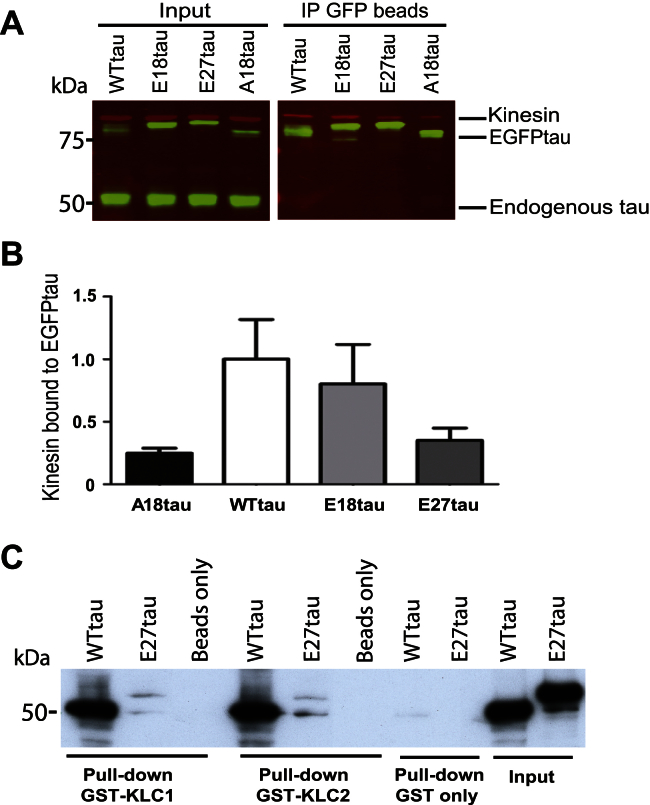
Tau pseudophosphorylation does not influence its binding to kinesin. (A) WTtau, E18tau, E27tau, or A18tau were expressed in primary rat cortical neurons and immunoprecipitated (IP) using an antibody to GFP. Total input and IP fractions were analyzed on Western blots probed with antibodies to kinesin heavy chain (red) and tau (green). (B) Kinesin/EGFPtau for each tau construct is expressed relative to WTtau. Values shown are mean ± standard error of the mean; *n* = 3; Student *t* test, relative to WTtau, showed no statistically significant differences. (C) Western blot of GST pull-down of WTtau and E27tau with GST-KLC1 and GST-KLC2, probed with tau antibody (TP70). Abbreviations: EGFP, enhanced green fluorescent protein; GFP, green fluorescent protein; GST, glutathione-S-transferase; KLC, kinesin light chain.

We have previously shown that in vitro phosphorylation of recombinant tau by GSK-3 increases the ability of tau to bind to the anterograde motor protein, kinesin-1 (Cuchillo-Ibanez et al., 2008). To determine the effect of tau pseudophosphorylation on its binding to kinesin-1 in neurons, primary cortical neurons were transiently transfected with WTtau or the tau phosphomutants. Cell lysates were incubated with GFP antibody linked to agarose beads, to pull down exogenous tau, and bound proteins were probed on Western blots with antibodies to tau or kinesin heavy chain (Fig. 5A). The relative amounts of kinesin and tau were quantified and expressed relative to WTtau (Fig. 5B). We found that although A18tau appeared to exhibit a trend toward reduced kinesin binding, this difference was not statistically significant (*p* = 0.077) and the phosphomimic tau mutants bound a similar amount of kinesin-1 as WTtau.

Because kinesin and tau bind microtubules, it is possible that tau could coprecipitate with kinesin through its binding to microtubules, rather than through a direct interaction with tau. Therefore, we investigated this interaction in vitro using beads containing GST alone, or fused to kinesin light chain (KLC) 1 or KLC2, to pull down recombinant WTtau or E27tau in the absence of microtubules. Beads linked to GST-KLC1 or GST-KLC2, but not GST only, pulled down substantial amounts of WTtau (Fig. 5C), however, very little E27tau bound to KLC under the same conditions. The fact that this result does not recapitulate the increased binding to kinesin observed with GSK-3-phosphorylated tau suggests that the tau phosphomimics might have some limitations as a model of direct phosphorylation for all tau functions (Combs et al., 2011). For example, E27tau harbors 5 glutamate serine subtitutions that are not phosphorylated by GSK-3 and it is possible that 1 or more of these mutated residues might negatively affect its association with kinesin. Interestingly, however, our findings eliminate the possibility that the increased number of motile particles of E27tau observed in axons, relative to WTtau (Fig. 4C and D), is because of enhanced kinesin binding by E27tau.

### 3.5 Exogenously expressed tau, but not endogenous tau, is degraded by the autophagic system

**Supplementary Fig. 1:**
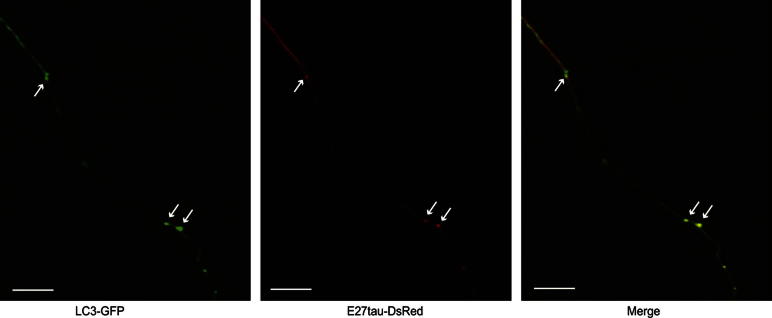
Colocalization of E27tau with LC3. Still image of neurons transfected with LC3-GFP and E27tau-DsRed. After 48 hours, time-lapse movies showed colocalization of E27tau with LC3 (arrows). Scale bar, 5 μm. Abbreviations: GFP, green fluorescent protein; LC3, light-chain 3.

**Supplementary Fig. 2:**
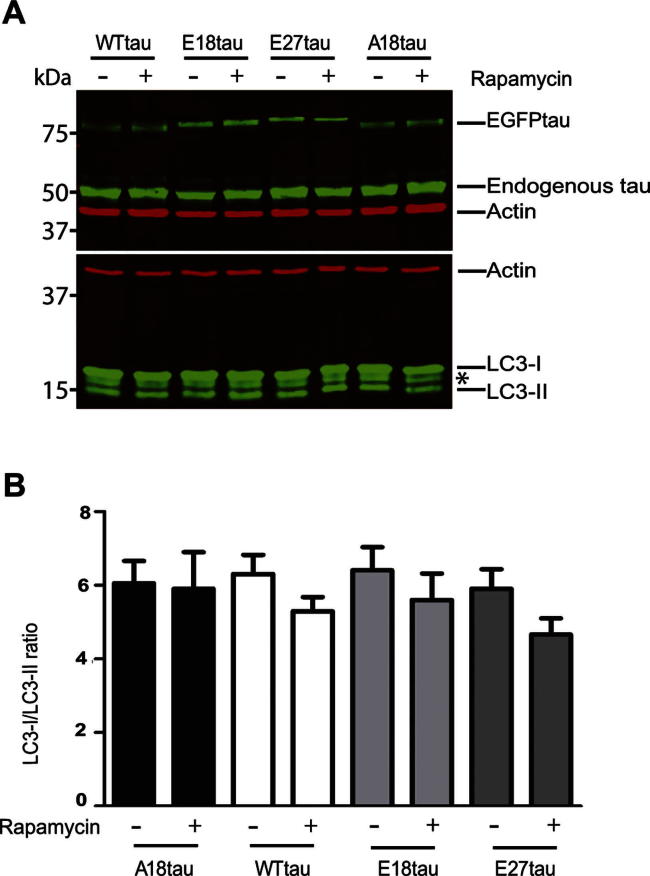
Lack of effect of rapamycin on tau degradation in neurons. Neurons were transfected with each tau construct and after 48-hour expression the cells were treated for 24 hours with 200 nM rapamycin to activate autophagy. (A) Total cell lysates were probed on Western blots using antibodies against tau (green), β-actin (red), and LC3 (green). * Nonspecific band detected by the LC3 antibody. (B) LC3-I/LC3-II was calculated for each condition (mean ± standard error of the mean; *n* = 3). Rapamycin did not increase LC3-II or reduce the LC3-I/LC3-II ratio as would be expected after activation of autophagy. Abbreviation: LC3, light-chain 3.

Autophagy has been implicated as a potentially important process in tau degradation, with some reports showing an influence of tau phosphorylation on autophagic clearance (Kim et al., 2011; Wang et al., 2009). To determine if some motile tau particles correspond to autophagosomes, cortical primary neurons were cotransfected with EGFP-LC3 (a marker of autophagosomes) and DsRed-E27tau. After 48-hour expression, we observed colocalization of E27tau with LC3 (Supplementary Fig. 1). To further investigate the effect of autophagy on tau degradation, we treated primary rat cortical neurons expressing WTtau, or the tau phosphomutants, with rapamycin (200 nM, 24 hours), to activate autophagy. In 7-DIV neurons, rapamycin treatment did not reduce the amounts of either endogenous or exogenous tau (Supplementary Fig. 2A). Furthermore, we found that rapamycin failed to activate autophagy because no change was detected in the ratio of the LC3 forms I and II (Supplementary Fig. 2B). These results are in agreement with a previous report in which rapamycin increased autophagic flux of endogenous tau in Neuro2A cells but not in primary cortical neurons (Kruger et al., 2012).

**Fig. 6:**
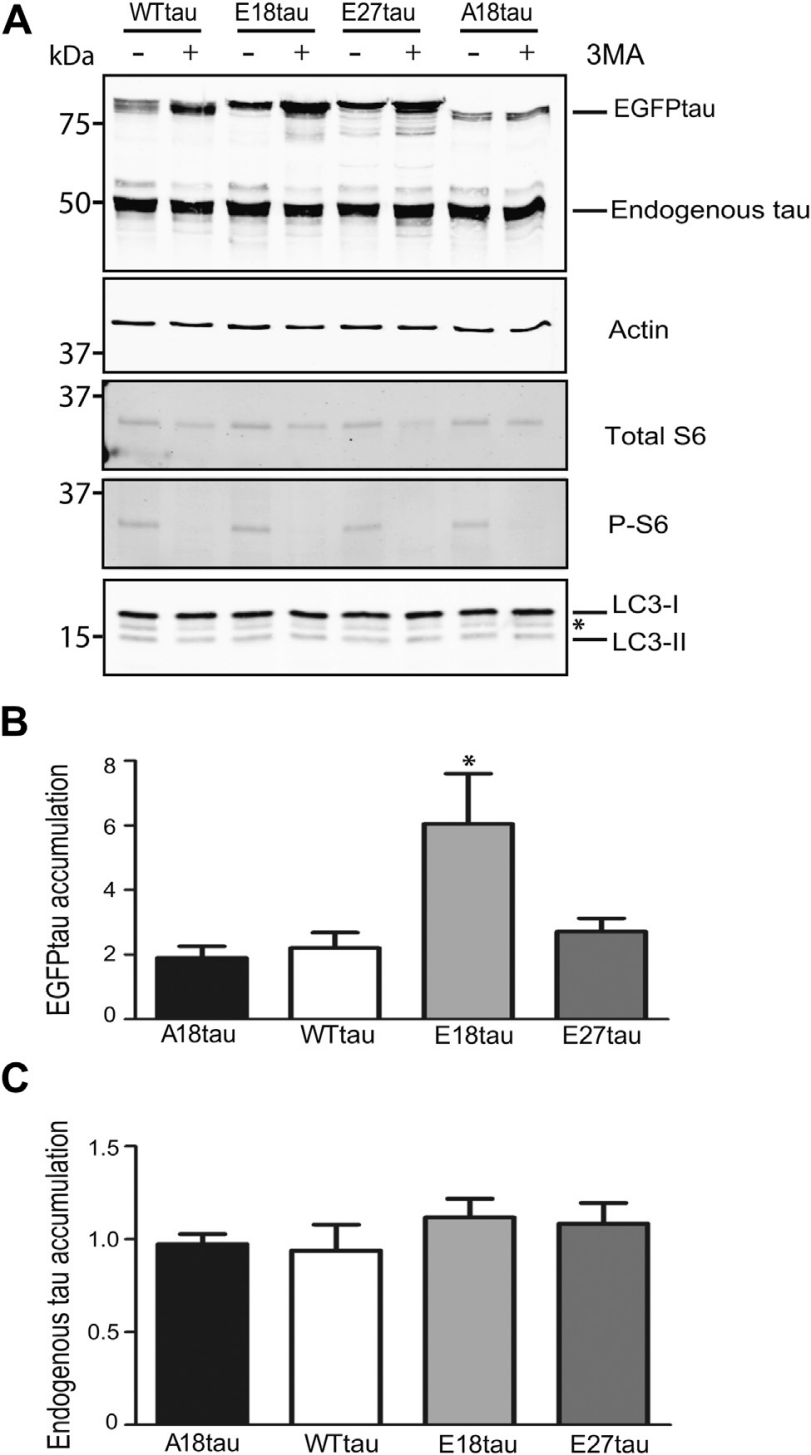
Inhibiting autophagy in cortical neurons results in differential degradation of tau phospho-mutants. Neurons were transfected with each tau construct and treated with 10 mM 3MA. (A) Cell lysates were analyzed on Western blots probed with antibodies to tau, β-actin, total and phosphorylated (P) ribosomal S6 protein, and LC3. * Nonspecific band detected by the LC3 antibody. (B) EGFPtau accumulation expressed as a ratio of 3MA-treated and untreated neurons, normalized to β-actin. Values shown are mean ± SEM; *n* = 5–6; 1-way analysis of variance. (C) Endogenous tau accumulation expressed as a ratio of 3MA-treated and untreated neurons, normalized to β-actin. Values shown are mean ± SEM; *n* = 6. Abbreviations: EGFP, enhanced green fluorescent protein; LC3, light chain 3; SEM, standard error of the mean.

We next treated rat cortical neurons expressing the different tau constructs with 3-methyladenine (3-MA, 10 mM, 24 hours), which blocks autophagy by targeting phosphoinositide 3-kinase (PI3K) (Blommaart et al., 1997; Petiot et al., 2000). Neuronal lysates were analyzed on Western blots probed with antibodies to tau, β-actin, total ribosomal protein S6, phosphorylated T-S6 (P-S6), and LC3 (Fig. 6A). Phosphorylated S6 is a surrogate marker of mammalian target of rapamycin activity, a key regulator of autophagy (Dann et al., 2007). We found that 3-MA effectively inhibits autophagy, confirmed by suppression of PI3K-mediated phosphorylation of S6 (Fig. 6A). Accumulation of exogenous tau was measured by calculating the ratio of 3-MA-treated to untreated neurons after normalizing to the amount of β-actin present (Fig. 6B). We found that inhibiting autophagy increased the amount of all the exogenously expressed tau forms, by 2–6-fold, without affecting endogenous tau (Fig. 6B). E18tau increased approximately 3-fold more than WTtau under conditions in which autophagy is inhibited by 3-MA (Fig. 6B; *p* < 0.05, 1-way analysis of variance). E27tau and A18tau also accumulated with 3-MA treatment, but these increases were similar to that of WTtau (Fig. 6B). These results indicate that inhibition of autophagy reduces degradation of all of the tau species examined, but that E18tau is affected more than the other tau constructs. However, we did not find any correlation (Spearman rank) between the number of moving tau particles and accumulation of tau because of inhibition of autophagy.

### 3.6 Proteasomal degradation of tau is modified by its phosphorylation state

**Fig. 7:**
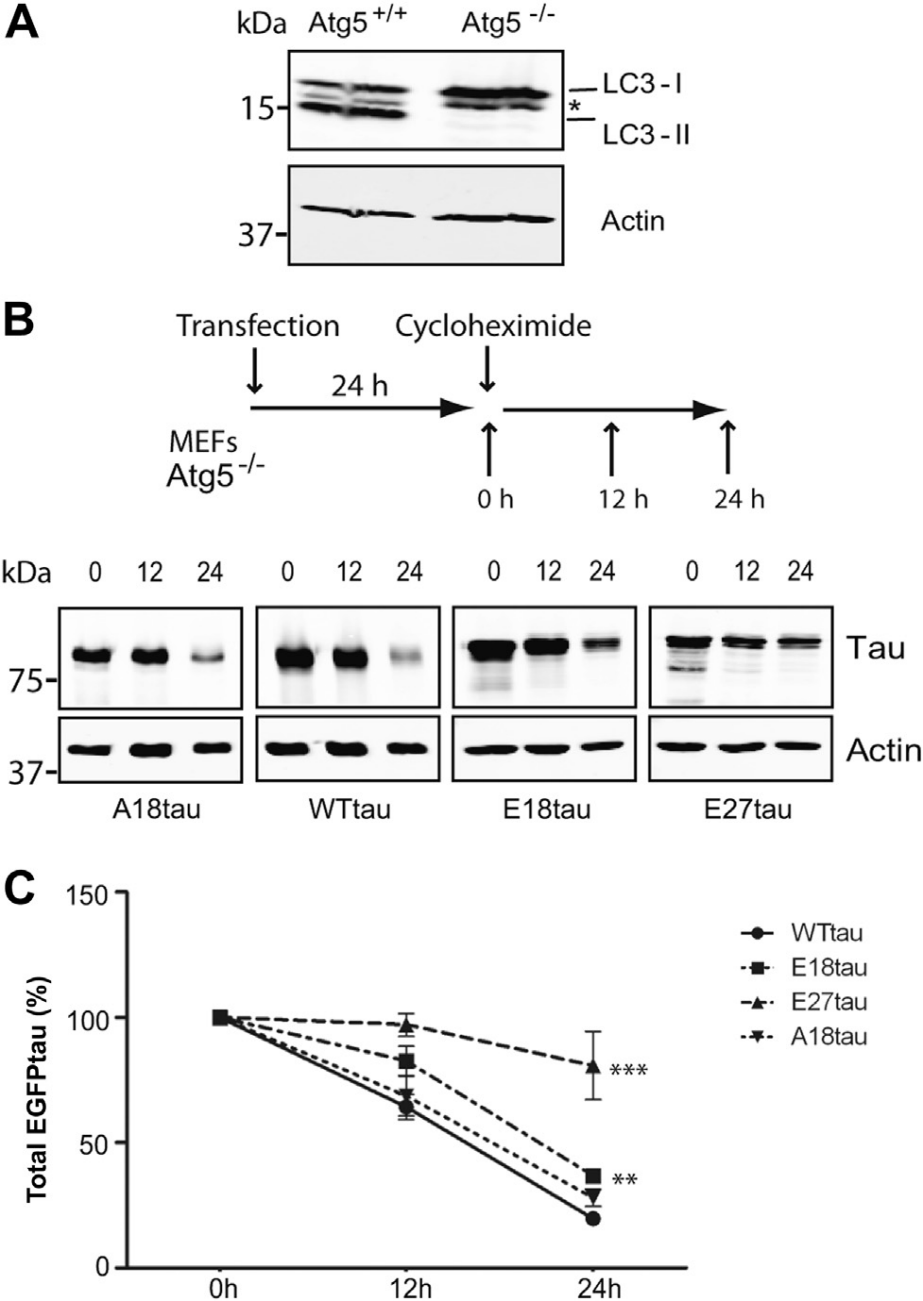
Autophagy-deficient MEFs exhibit differential degradation of tau phospho-mutants. (A) Western blot of Atg5^+/+^ and Atg5^-/-^ MEFs probed with antibodies to LC3 and β-actin. * Nonspecific band detected by the LC3 antibody. (B) Atg5^-/-^ MEFs were transfected with each tau construct. After 24 hours, cells were treated with 100 μM cycloheximide and harvested at 0, 12, and 24 hours. Cell lysates were analyzed on Western blots probed with antibodies to tau (DAKO) and β-actin. (C) The amount of tau remaining after cycloheximide treatment for 12 and 24 hours was calculated as the percentage of tau present at 0 hours. Values shown are mean ± standard error of the mean; *n* = 6; ** *p* < 0.01, *** *p* < 0.001, Student *t* test, relative to WTtau at 24 hours. Abbreviations: EGFP, enhanced green fluorescent protein; LC3, light chain 3; MEF, mouse embryonic fibroblasts.

In addition to autophagy, tau can also be degraded by the UPS and inhibition of the UPS results in increased amounts of tau in SHSY-5Y cells (David et al., 2002) and rat brain (Liu et al., 2009). We therefore examined the contribution of the UPS to tau degradation in autophagy-deficient MEFs lacking Atg5. To confirm the lack of autophagy in these cells, lysates of control Atg5^+/+^ and autophagy-deficient Atg5^-/-^ cells were probed on blots with an antibody to LC3. As expected, LC3-I was present in both cell lines, but LC3-II was undetectable in Atg5^-/-^ MEFs because of the absence of autophagy (Fig. 7A).

**Supplementary Fig. 3:**
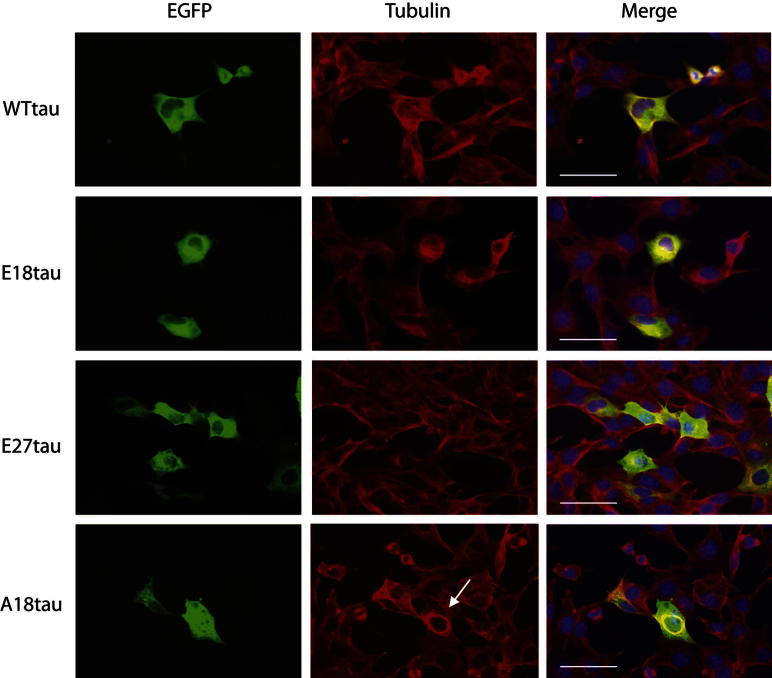
Wild type and phosphomutant tau species do not aggregate in autophagy-deficient MEFs. WTtau, E18tau, E27tau, and A18tau were expressed in transfected autophagy-deficient (Atg5^-/-^) MEFs. Tubulin was stained using DM1A antibody and tau was detected by EGFP fluorescence. No tau aggregates were detected after 48-hour expression. A18tau exhibited a greater ability to bundle microtubules (white arrow). Scale bar, 50 μm. Abbreviations: EGFP, enhanced green fluorescent protein; MEF, mouse embryonic fibroblast cell.

Atg5^-/-^ autophagy-deficient MEFs were transfected with constructs expressing WTtau or the 3 tau phosphomutants. Twenty-four hours after transfection, cells were treated with 100 μM cycloheximide, to block protein synthesis, and cells were harvested immediately, 12 hours, and 24 hours after treatment (Fig. 7B). The absence of autophagy in MEFs did not lead to aggregation of any of the tau species after 48-hour expression (Supplementary Fig. 3). Atg5^-/-^ MEFs expressing tau were lysed and analyzed on Western blots using antibodies to tau and β-actin (Fig. 7B). The amount of tau present at each time point was quantified and expressed relative to the amount of tau present at 0 hours (Fig. 7C). We found that, in comparison with WTtau, the amounts of E18tau and E27tau were increased at 24 hours, indicating that the 2 phosphomimics exhibited reduced rates of degradation by the UPS, compared with WTtau (Student *t* test, *p* < 0.05). The estimated half-lives for WTtau, A18tau, E18tau, and E27tau in MEFs were approximately 16, 18, 21, and >24 hours, respectively. These results are in line with those obtained from 3-MA-treated neurons expressing E18tau (Fig. 6), showing that E18tau is more likely than WTtau to be degraded by the autophagic route. However, E27tau did not accumulate in 3-MA-treated neurons compared with WTtau, and exhibited a reduced degradation rate by the UPS in MEFs. A possible explanation for this difference between the 2 tau phosphomimics could be that the 9 additional glutamate substitutions in E27tau might cause the protein to adopt a conformation which affects its recognition by the degradation machinery.

### 3.7 Proteasomal degradation of tau is minimal in cortical neurons

To test if the proteasome system has a role in tau degradation in neurons, we used the same approach of inhibiting protein synthesis with cycloheximide to measure the rate of tau degradation in primary neurons. Cycloheximide causes an early suppression of autophagy because this process requires the involvement of multiple short-lived proteins that are essential for early autophagosome formation (Lawrence and Brown, 1993). Thus, in neurons treated with cycloheximide, the UPS, but not autophagy, remains effective, and hence the contribution of the proteasome to tau degradation can be measured.

**Fig. 8:**
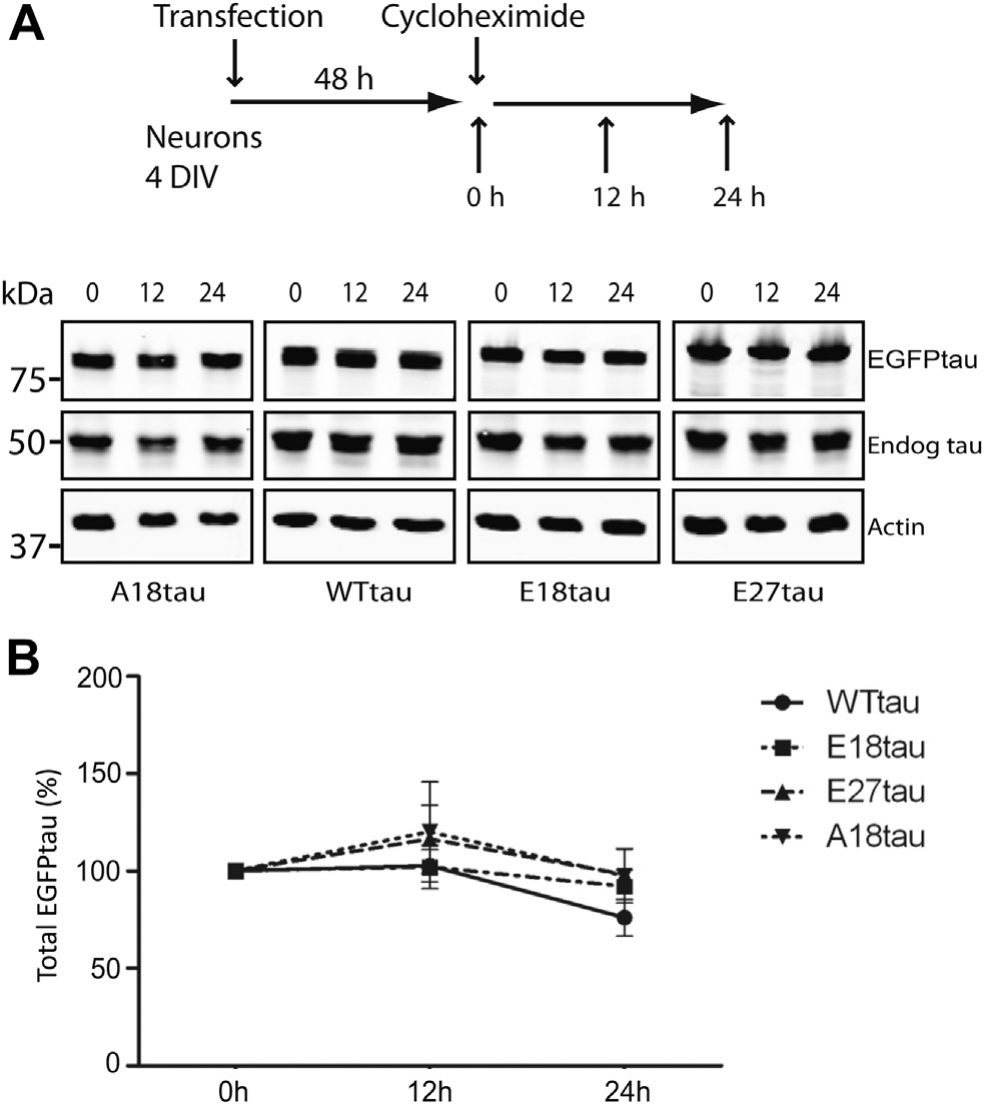
Lack of tau degradation by the proteasome in neurons. (A) Primary rat cortical neurons were transfected with each tau construct. After 48 hours, neurons were treated with 100 μM cycloheximide and harvested at 0, 12, and 24 hours. Neuronal lysates were analyzed on Western blots probed with antibodies to tau (DAKO) and β-actin. (B) The amount of EGFPtau remaining after cycloheximide treatment for 12 and 24 hours was calculated as the percentage of tau present at 0 hours. Values shown are mean ± standard error of the mean; *n* = 6. Abbreviations: DIV, days in vitro; EGFP, enhanced green fluorescent protein; Endog, endogenous.

Rat primary cortical neurons exogenously expressing each of the 4 tau species, were treated with 100 μM cycloheximide 48 hours after transfection and cells were harvested immediately, and 12 and 24 hours after treatment (Fig. 8A). Neuronal lysates were analyzed on Western blots using antibodies to tau and β-actin, and the amount of tau remaining at 12 and 24 hours was calculated as the percentage of EGFP-tau present at 0 hours (Fig. 8A). Under conditions of autophagy inhibition and an active UPS, endogenous and exogenous tau exhibited very low rates of degradation in neurons compared with MEFs. Thus, the amount of endogenous tau remaining in neurons was similar at 0, 12, and 24 hours after application of cycloheximide (Fig. 8B). The half-lives of all of the exogenously expressed tau species in neurons exceeded 24 hours and only minimal tau degradation was observed at this time point. These results demonstrate that the UPS has a very limited capability to degrade endogenous tau in cultured cortical neurons and hence tau turnover is very low under these conditions.

## 4 Discussion

Here we have investigated the effect of tau phosphorylation on its rate of axonal transport and degradation. The tau phosphomimics, E18tau, and E27tau (Goedert et al., 1989; Smith et al., 2000), harbor glutamate substitutions of serine and threonine residues that correspond to many of those targeted by GSK-3, a candidate kinase for tau phosphorylation (Hanger et al., 2007). Thus, 16 sites in E18tau and 22 sites mutated in E27tau are reported to be targets of GSK-3 phosphorylated tau, suggesting that these constructs might represent reasonable mimics of phosphorylated tau when expressed in cells.

Ectopic expression of tau in a variety of cell types results in the appearance of characteristic bundles of microtubules, presumed to result from increased tubulin stabilization (Kanai et al., 1989; Langkopf et al., 1995; Lee and Rook, 1992; Scott et al., 1992; Stoothoff et al., 2008; Takemura et al., 1992). Therefore, we first established the microtubule bundling properties of the tau phosphomutants in CHO cells. Though untransfected cells exhibit an ordered microtubular array, WTtau induces the formation of bundles of microtubules that frequently appeared as a thick ring encircling the inner periphery of the cell. E18tau and E27tau expression did not alter either the size or morphology of CHO cells, and microtubule bundles were rarely observed, even in cells highly expressing the phosphomimic tau constructs. In contrast, even low expression of A18tau caused microtubules to bundle very effectively. E18tau and E27tau bind less well to microtubules compared with WTtau and, conversely, A18tau showed increased microtubule binding. These findings are in good agreement with our observations of the effects of these tau variants on microtubule bundling, and also with previous reports of reduced microtubule binding by phosphorylated tau (Bramblett et al., 1993; Busciglio et al., 1995; Wagner et al., 1996). Our data support the view that a greater proportion of highly phosphorylated tau might be detached from microtubules in neurons compared with tau phosphorylated to a low stoichiometry. Lack of binding of phosphorylated tau to microtubules would effectively increase cytoplasmic tau, which could result in the formation of intracellular tau aggregates in the tauopathies.

We examined the axonal transport kinetics of tau because regulation of microtubule binding and tubulin stabilization are important for the axonal transport of multiple cargoes (Ebneth et al., 1998; Mandelkow et al., 2003). Interestingly, compared with WTtau, we observed significantly more motile particles of phosphomimic tau in individual axons, and this correlated with the degree of tau phosphorylation (*p* < 0.05). However, phosphomimic tau particles are indistinguishable from WTtau with respect to their velocity and the number and duration of pauses. This finding suggests that, when the molecular motor complex binds to tau, its phosphorylation state does not influence transport kinetics. We did not detect any movement of the nonphosphorylatable A18tau, possibly because of its increased microtubule binding. Notably, we found that coexpression of WTtau and E27tau in neurons resulted in their codistribution, although a proportion of E27tau puncta did not overlap with the generally more diffuse axonal WTtau. A plausible explanation for this finding is that phosphorylated tau has a reduced ability to bind to microtubules, which could result in accumulation of E27tau.

The increased number of motile phosphomimic tau particles observed in the axonal transport analysis led us to investigate changes in tau degradation that could explain the increase of pseudophosphorylated tau protein. The autophagic and proteasome systems have been linked to tau degradation and we decided to study the contribution of both pathways to tau accumulation. Autophagosome dynamics in primary neurons has been described by Maday et al. (2012), who found that autophagosome biogenesis occurs distally in the axon. Autophagosomes switch from an initial bidirectional movement to unidirectional movement toward the cell soma driven by dynein at a mean speed of 0.45 μm/s. Thus, the puncta comprised of solely E27tau in our coexpression experiment could represent autophagosomes undergoing retrograde transport, a view supported by our finding of colocalization of E27tau with the autophagic marker, LC3.

The role of autophagy in neurodegeneration is controversial, with some reports concluding that inhibiting autophagy is neuroprotective (Sadasivan et al., 2010; Wang et al., 2011), and other studies showing that activating autophagy, using rapamycin, trehalose, or methylene blue, reduces aggregated proteins and improves neuronal survival (Berger et al., 2006; Congdon et al., 2012; Renna et al., 2010; Sarkar and Rubinsztein, 2008). Interestingly, a recent study of mutant P301S tau transgenic mice has shown that enhancing autophagy reduces tau aggregates, decreases insoluble tau, and improves neuronal survival (Schaeffer et al., 2012). In agreement with a previous report, we were unable to detect an effect of rapamycin in primary cortical neurons (Kruger et al., 2012) and endogenous tau did not accumulate in neurons after inhibition of autophagy with 3-MA (Kim et al., 2011; Wang et al., 2009, 2010). These results are in agreement with a previous study examining tau degradation in rat brain (Zhang et al., 2009). Taken together, these results suggest that autophagy is unlikely to be a major contributor to degradation of endogenous tau in neurons.

In contrast to the lack of effect of autophagic inhibition on endogenous tau, exposure of neurons to 3-MA resulted in the accumulation of exogenously expressed wild type and phosphomutant tau. 3-MA induced the accumulation of all forms of exogenously expressed tau, with E18tau increasing 3-fold relative to WTtau. This finding implies that there might be conformational differences between the 2 tau phosphomimics, which could result in differential recognition of phosphorylated forms of tau as a substrate for degradation. Our findings support and extend reports of the effects of 3-MA on wild type and phosphomutant tau in cells (Kim et al., 2011; Wang et al., 2009) and suggest that elevated phosphorylation could enhance tau degradation by the autophagic pathway.

To determine the effect of the proteasome on tau in neurons, in conditions in which autophagy is inhibited, we treated cultured neurons with cycloheximide to prevent synthesis of proteins involved in early autophagosome formation and hence measure primarily proteasomal degradation. In conditions when autophagy is inhibited, tau degradation was minimal after 24 hours, in line with its reported half-life of approximately 60 hours in HT22 cells (Poppek et al., 2006). All forms of exogenous tau were degraded at a similar rate to endogenous tau, with exogenously expressed phosphomutant tau appearing to be degraded slightly slower than WTtau, although this difference was not significant. Though our results are suggestive that increased phosphorylation might reduce the rate of tau degradation, this could not be determined conclusively because of the slow overall rate of tau degradation in neurons.

Interestingly, the effect of autophagy inhibition using 3-MA is not recapitulated in neurons expressing the phosphomutants and treated with cycloheximide, which also inhibits autophagy. A possible explanation for this is that cycloheximide inhibition of autophagy might be secondary to blockage of protein synthesis (Lawrence and Brown, 1993). Treatment of neurons with 3-MA results in the specific blocking of autophagy through PI3K inhibition. Another possibility is the potential cross-talk between autophagy and the UPS in neurons in which inhibition of autophagy could also decrease the efficiency of the UPS (Korolchuk et al., 2009). Blocking autophagy has been shown to lead to an increase in proteasomal substrates, which can be explained by the accumulation of p62, resulting in delayed delivery of ubiquinated proteins to the proteasome (Korolchuk et al., 2009).

The effect of proteasome inhibition on tau in cultured embryonic neurons is also somewhat controversial. Inhibiting the proteasome has been reported not to affect the amount of tau in neuronal cells (Brown et al., 2005). In contrast, when tau is exogenously expressed in HT22 cells, phosphorylation inhibits its degradation by the proteasome (Poppek et al., 2006). Proteasome inhibition in vivo also increases the amount of tau in rat brain, suggesting that this mechanism might have a role in regulating turnover of endogenous tau (Liu et al., 2009). One possible explanation for these contradictory findings could be the widespread use of MG132 as a proteasome inhibitor. MG132 has pleiotropic effects that include: (1) modifying cytomegalovirus promoter expression, which could affect exogenous tau expression in some studies (Biasini et al., 2004); (2) activation of calpain, which could result in increased tau cleavage (Hamano et al., 2009); and (3) induction of autophagy (Du et al., 2009; Klappan et al., 2012; Zheng et al., 2011). This highlights the difficulty of studying the effect of a specific proteolytic system in cells because cross-talk and potential compensation between autophagy and the proteasome have been reported (Korolchuk et al., 2010; Zheng et al., 2009). To eliminate these confounding factors, we examined tau degradation in autophagy-deficient MEFs, which lack Atg5, a gene involved in autophagosome formation (Kuma et al., 2004). Atg5^-/-^ MEFs are dependent for proteolysis on cellular mechanisms other than autophagy, and proteins are therefore eliminated by the proteasome and/or by calpain-mediated degradation. Even when highly expressed, WTtau, E18tau, E27tau, and A18tau did not form aggregates in Atg5^-/-^ MEFs, suggesting that inhibition of autophagy might not have a marked effect on tau accumulation in these cells. However, there was a significant reduction in the rate of degradation of the tau phosphomutants compared with WTtau, indicating that increased phosphorylation might reduce tau degradation by the UPS and/or calpain, possibly directing tau toward an autophagic route.

Extrapolation of these results to neurons undergoing neurodegeneration, in which tau phosphorylation plays a key role, suggests a mechanism whereby highly phosphorylated tau could result in abnormal accumulation of cytoplasmic tau and a cascade of secondary events that might contribute to neurotoxicity and cell death. For example, increased steady-state amounts of phosphorylated cytoplasmic tau could overload kinesin-based molecular motor transport mechanisms, thereby blocking or reducing the movement of other cargoes, including mitochondria and vesicles. Thus, it is plausible that increased axonal transport could contribute to the mislocalization and accumulation of tau, and thereby participate in the spreading of tau pathology in neurodegenerative disease. Secondly, increased phosphorylation could lead to the removal of tau from the plasma membrane, and potentially to disruption of intracellular signaling pathways (Brandt et al., 1995; Pooler and Hanger, 2010; Pooler et al., 2012). Finally, binding of tau to the plasma membrane and to microtubules could be essential for autophagic flux because the plasma membrane is a major source of autophagosomal components (Cuervo, 2010; Ravikumar et al., 2010), and autophagy is dependent on microtubule integrity (Aplin et al., 1992; Webb et al., 2004).

We conclude that in autophagy-deficient mouse embryonic fibroblasts, but not in neurons, proteasomal degradation of phosphomutant tau is reduced compared with wild type tau. Autophagic and proteasomal pathways are involved in tau degradation, with autophagy appearing to be the primary route for clearing phosphorylated tau in neurons. Enhancing the autophagic pathway might have potential as a novel therapeutic approach for the removal of damaging, highly phosphorylated and aggregated proteins in Alzheimer's disease and other neurodegenerative disorders.

## Disclosure statement

The authors declare no conflicts of interest.

All animal work in this study was performed following UK Home Office guidelines.

## Figures and Tables

**Supplementary Fig. 4:**
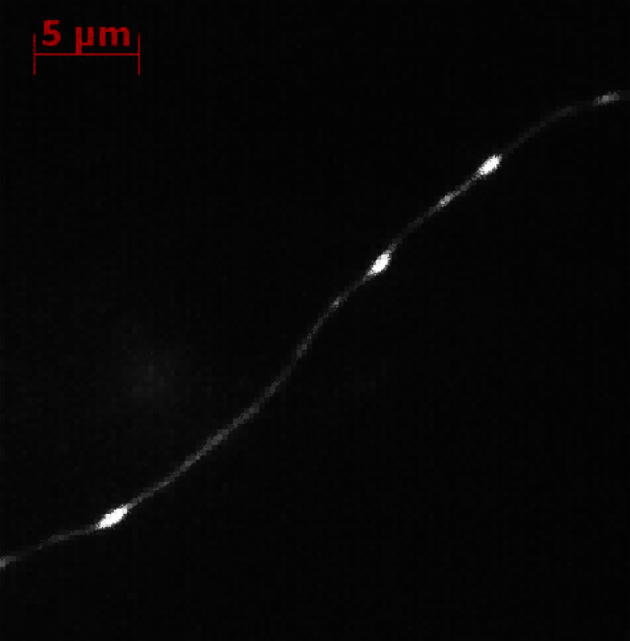


**Supplementary Fig. 5:**
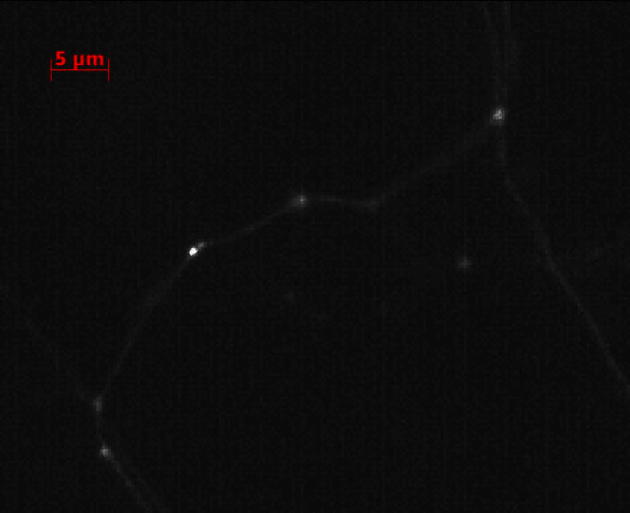


**Supplementary Fig. 6:**
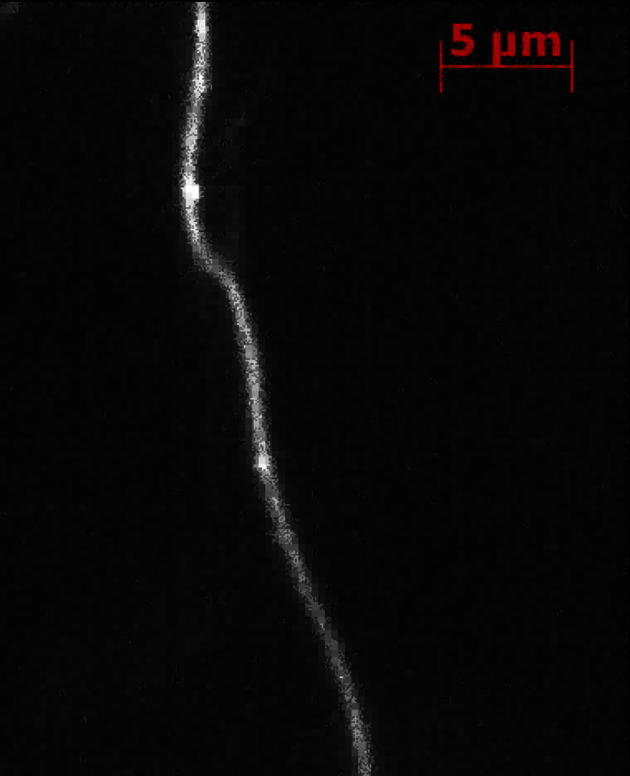


## Appendix A Supplementary data

Video 1 An individual motile WTtau particle.

Video 2 Two E18tau particles moving in the same axon.

Video 3 Three E27tau particles moving in the same axon.
