# Supplementary figures and images for: Tau phosphorylation affects its axonal transport and degradation
Source: Neurobiol Aging. 2013 Sep;34(9):2146–57. doi: 10.1016/j.neurobiolaging.2013.03.015 (PMC3684773; doi:10.1016/j.neurobiolaging.2013.03.015)

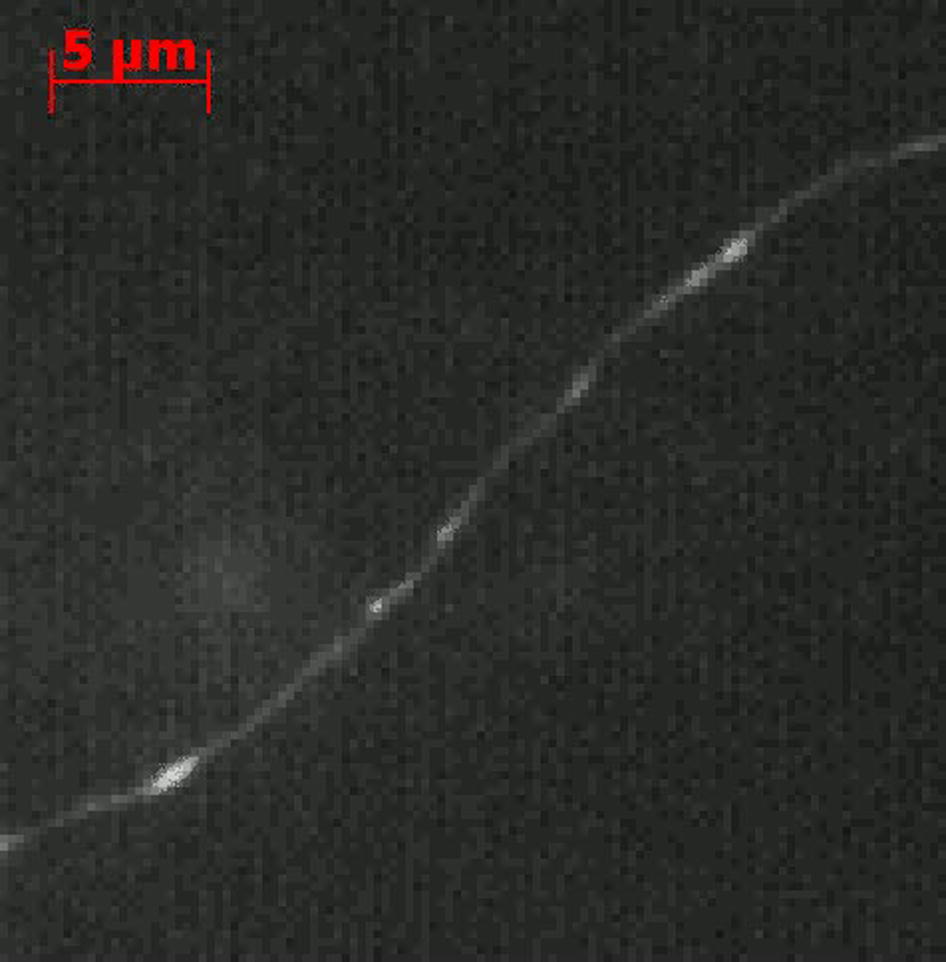

Supplement: Video 2 — Two E18tau particles moving in the same axon. [file mmc2.jpg]
